# Use of systemic hormonal contraception and depression: a nested case-control study

**DOI:** 10.1192/j.eurpsy.2023.257

**Published:** 2023-07-19

**Authors:** E. Toffol, T. Partonen, O. Heikinheimo, A. Latvala, A. But, J. Haukka

**Affiliations:** 1 Department of Public Health, University of Helsinki; 2 Finnish Institute for Health and Welfare; 3 University of Helsinki, Helsinki, Finland

## Abstract

**Introduction:**

Depression is twice as common in women as in men, especially in the young age group. Multiple factors may contribute to this gender difference. Growing attention is being focused on the role of sex hormones, including those of hormonal contraception (HC). Some recent studies have indicated a higher risk of depression among women using HC, although the results are inconclusive.

**Objectives:**

The aim of this study is to examine the associations between the use of hormonal contraception and the risk of depression in childbearing age women.

**Methods:**

The original cohorts for the study included all women aged 15-49 years with at least one redeemed prescriptions for HC in Finland in 2017 (n=294,356), and a 1:1 age-matched cohort of non-users. After exclusion of prevalent cases (n=35,102), all incident cases of depression (as recorded in the Care Register of Health Care and Register of Primary Health Care Visits) in 2018-2019 were identified (n=23,480), and a 4:1 age-matched control group (n=93,920) was selected from the above cohorts. Current use of HC in the 180 days before the event was compared in cases and controls, and associations with risk of depression were tested via conditional multivariate logistic regression models.

**Results:**

During the follow-up, 23,480 incident cases of depression were identified. Current use (in the 180 days before the event) of HC (OR 0.82, 95% CI 0.79-0.85), in particular of estradiol- or ethinylestradiol-containing combined HC was associated with a lower risk of depression (OR 0.83, 95% CI 0.76–0.89; OR 0.74, 95% CI 0.71–0.78, respectively) compared to non-use of HC. The results remained significant (OR 0.87, 95% CI 0.81-0.95; and OR 0.77, 95% CI 0.73-0.81, respectively) after controlling for covariates (marital and socioeconomic status, education level, chronic diseases). Use of progestin-only contraception was not associated with altered risk of depression.

**Conclusions:**

Use of HC in childbearing age women is not associated with increased risk of depression. Rather, the use of estradiol- or ethinylestradiol-containing HC is associated with a lower risk of depression.

**Disclosure of Interest:**

None Declared

